# Essential Oil Composition of* Pinus peuce* Griseb. Needles and Twigs from Two National Parks of Kosovo

**DOI:** 10.1155/2016/5393079

**Published:** 2016-08-08

**Authors:** Avni Hajdari, Behxhet Mustafa, Dashnor Nebija, Hyrmete Selimi, Zeqir Veselaj, Pranvera Breznica, Cassandra Leah Quave, Johannes Novak

**Affiliations:** ^1^Department of Biology, Faculty of Mathematical and Natural Sciences, University of Prishtina, Mother Theresa Street, 10000 Prishtinë, Kosovo; ^2^Institute of Biological and Environmental Research, Faculty of Mathematical and Natural Sciences, University of Prishtina, Mother Theresa Street, 10000 Prishtinë, Kosovo; ^3^Department of Pharmaceutical Chemistry, Faculty of Medicine, University of Prishtina, Mother Theresa Street, 10000 Prishtinë, Kosovo; ^4^Faculty of Education, University of Prishtina “Hasan Prishtina”, Mother Teresa, 1000 Prishtinë, Kosovo; ^5^Center for the Study of Human Health, Emory University, 550 Asbury Circle, Candler Library 107E, Atlanta, GA 30322, USA; ^6^Department of Dermatology, Emory University School of Medicine, 1518 Clifton Road NE, CNR 5035, Atlanta, GA 30322, USA; ^7^Institute of Animal Nutrition and Functional Plant Compounds, University of Veterinary Medicine, Veterinärplatz 1, 1210 Vienna, Austria

## Abstract

The principal aim of this study was to analyze the chemical composition and qualitative and quantitative variability of essential oils obtained from seven naturally grown populations of the* Pinus peuce* Grisebach, Pinaceae in Kosovo. Plant materials were collected from three populations in the Sharri National Park and from four other populations in the Bjeshkët e Nemuna National Park, in Kosovo. Essential oils were obtained by steam distillation and analyzed by GC-FID (Gas Chromatography-Flame Ionization Detection) and GC-MS (Gas Chromatography-Mass Spectrometry). The results showed that the yield of essential oils (*v/w* dry weight) varied depending on the origin of population and the plant organs and ranged from 0.7 to 3.3%. In total, 51 compounds were identified. The main compounds were *α*-pinene (needles: 21.6–34.9%; twigs: 11.0–24%), *β*-phellandrene (needles: 4.1–27.7; twigs: 29.0–49.8%), and *β*-pinene (needles: 10.0–16.1; twigs: 6.9–20.7%). HCA (Hierarchical Cluster Analysis) and PCA (Principal Component Analyses) were used to assess geographical variations in essential oil composition. Statistical analysis showed that the analyzed populations are grouped in three main clusters which seem to reflect microclimatic conditions on the chemical composition of the essential oils.

## 1. Introduction

Essential oils represent an important class of constituents among various plant families. Due to the high content and interesting composition of their essential oils, numerous species of the Pinaceae family have been traditionally used in medicine, cosmetics, and the food industry. The essential oils of* Pinus* species demonstrate primarily antimicrobial, expectorant activities and promote blood circulation [1–3]. Herbal medicines containing essential oils from* Pinus* spp. are therapeutically used externally or by inhalation to treat different medical conditions, including respiratory disease, common colds, and rheumatic complaints such as muscle and joint pain [1–3]. Antioxidative, free radical scavenging, insecticidal, phytotoxic, larvicidal, repellent, anti-inflammatory, antiviral, and antifungal properties of* Pinus* essential oils have been reported as well [4–12]. Pine oil has also been used as a component of aromatherapy recipes [13]. In the European Pharmacopoeia, Ph.Eur.7.0 [14], monographs for the essential oils of the most prominent representatives of this family, namely,* Pinus sylvestris* and* Pinus mugo,* are published. Turpentine oil from* Pinus pinaster* is officinal in European Pharmacopoeia, Ph.Eur. 6.2 [15].* Pinus peuce*, also known as Balkan pine or Macedonian pine, is a Balkan endemic conifer tree growing in mountains of Bulgaria, Serbia, Macedonia, Greece, Kosovo, and Montenegro between ca. 600 and 2200 m.a.s.l. [16]. The mature tree may reache up to 36–42 m and the trunk diameter is 60–80 cm, but in certain individuals it may be up to 120 cm [16, 17].

Phytochemical analysis including chemical composition of essential oils obtained from* Pinus peuce* and its biological activity has been addressed in recently published papers [2, 18–20]. GC and GC-MS were used to study composition of essential oils of* P. peuce* in oil samples isolated from shots and cones [21, 22]. In addition HPLC, hyphenated with ESI-MS, was used to investigate the flavonoid content in* Pinus peuce* [23]. The n-alkane composition and the nonacosane-10-ol content in the needle waxes of different* Pinus peuce* populations and between* Pinus peuce*,* Pinus heldreichii,* and* Picea omorica* have been compared [24, 25]. Furthermore, genome size and base composition of* Pinus* species from the Balkan region hasve been estimated by flow cytometry [26], and isozyme variation in* Pinus peuce* has been studied by Zhelev et al. [27]. Results of chemical composition (terpenes and n-alkanes) of some populations of* P. peuce* from Kosovo had already been investigated ([2, 19–22] and older references cited therein).

The principal aim of our study is to elucidate the chemical composition of essential oils obtained from needles and twigs of* P. peuce* naturally grown in Kosovo and to assess the variation of the content and chemical composition of the essential oils among different locations and different plant organs.

## 2. Material and Methods

### 2.1. Plant Material

Plant material of* Pinus peuce* was collected from July to September 2013 in seven different populations growing wild in Kosovo. Three populations originated from the Sharri National Park whereas other three were coming from the Bjeshkët e Nemuna National Park (Table 1). Two to four replicate samples of needles and twigs were analyzed, and each sample was gathered from 3 to 4 individual plants from each population.

Samples were distilled and analyzed separately. Voucher specimens of each population were deposited in the Herbarium of the Department of Biology, University of Prishtina (Table 1).

### 2.2. Essential Oil Isolation

Plant material was air-dried in the shade at room temperature and cut into small pieces (<0.5 cm). Separated needles and twigs (only woody parts) were subjected to essential oil distillation. For distillation, 50 g of dry tissue was placed into 0.5 liter of water in a 1 liter flask and distilled at a rate of 3 mL/min in a Clevenger apparatus for 3 h. The samples were stored in the dark at −18°C in the freezer pending further analysis. The yield of essential oil is expressed as a volume/weight percentage, accounted to the air-dried plant material (%* v/w* of dried material).

### 2.3. GC and GC-MS Analyses

GC/FID analyses were performed using an Agilent 7890A gas chromatograph (Agilent Technologies) equipped with the flame ionization detector (FID). The separation was conducted on a HP-5MS column 30 m × 0.25 mm with 0.25 *μ*m film thickness. Helium was used as carrier gas with an initial flow rate of 0.6 mL/min and subsequently at a constant pressure of 12.0 psi. The front inlet was maintained at 250°C in a split ratio of 50 : 1. The GC oven temperature increased from 60°C to 260°C at a rate of 5°C/min and the FID operated at 250°C with an air flow of 350 mL/min and a hydrogen flow of 35 mL/min. The injection volume was 1.0 *μ*L.

GC/MS analyses were performed using an Agilent 7890A GC system coupled to a 5975C MSD (Agilent Technologies). The ionization energy was 70 eV with a mass range of 40–400* m/z*. The separation was conducted with the same column and temperature program as for the analytical GC.

The Kovats Retention Indexes were experimentally determined and compared with those from the literature [28]. The calculation of the Kovats indices was made based on a linear interpolation of the retention time of the homologous series of n-alkanes (C9–C29) under the same operating conditions. The components were also identified by comparing the mass spectra of each constituent with those stored in the NIST 08.L and WILEY MS 9th database and with mass spectra from the literature [28]. The percentage ratio of essential oils components was computed by the normalization method of the GC/FID peak areas.

### 2.4. Statistical Analysis

Hierarchical Cluster Analysis (HCA) and Principal Component Analyses (PCA) were used to evaluate whether the identified essential oils components can be useful for reflecting the population diversity of* P. peuce*. PCA and HCA analyses were performed using the statistical analysis software, XLSTAT Version 2014.2.03 (STATCON, Witzenhausen, Germany). The oil components with concentrations higher than 1% of the total oil amount in twigs and/or needles were subjected to statistical analyses (Figures 2 and 3).

## 3. Results and Discussion

The chemical composition of essential oils obtained from needles and twigs of* Pinus peuce* grown in three populations in Sharri National Park and four populations in Bjeshkët e Nemuna National Park are presented in Tables 2 and 3, respectively.

Experimental data revealed that the essential oils were mainly composed of monoterpenes. Their concentration in twigs was higher in comparison to the needles. As presented in Tables 2 and 3, the concentration of monoterpenes in samples obtained from needles and originating from the Sharri National Park ranged from 62.6 to 73.1% and in twigs from 74 to 78.5% whereas, in samples originated from Bjeshkët e Nemuna National Park, the concentrations of monoterpenes in needles and twigs ranged from 59.4 to 65.5% and 71.8 to 82.9%, respectively. Monoterpenes were followed by sesquiterpenes and their respective concentrations in needles and twigs samples originated from the Sharri National Park ranged from 10.7 to 15.6% and 9.9 to 13.2%, while in samples originating from the Bjeshkët e Nemuna National Park the concentrations of sesquiterpenes in needles and twigs ranged from 13.8 to 27.1% and 9.3 to 14.5%, respectively. Oxygenated monoterpenes concentrations in needles and twigs were 9.0–14.3% and 4.7–5.6%, respectively, in the Sharri National Park and 1.6–16.9% and 0.9–3.7%, respectively, in the Bjeshkët e Nemuna National Park. Oxygenated sesquiterpenes were less abundant than the previous groups (concentrations in needles and twigs: 2.7–4.1% and 2.8–3.5%, resp., in Sharri National Park and 3.6–5.9% and 2.1–6.3%, resp., in Bjeshkët e Nemuna National Park). Volatile diterpenes were less abundant constituents in the samples of both populations with <1%.

In samples obtained from Sharri National Park and Bjeshkët e Nemuna National Park the dominant constituents were the monoterpenes *α*-pinene, *β*-phellandrene, *β*-pinene, camphene, and bornyl acetate, as well as the sesquiterpene germacrene.

In the samples obtained from needles of* P. peuce* from the Sharri National Park, *α*-pinene with an average concentration of 31.6%, was dominant, followed by *β*-pinene (13.8%), *β*-phellandrene (9.8%), germacrene D (9.2%), camphene (7.7%) and bornyl acetate (4.4%), while in needles originating from the Bjeshkët e Nemuna National Park the order of the compounds was *α*-pinene (29.0%) followed by germacrene D (16.6%), *β*-phellandrene (11.4%), *β*-pinene (10.9%), bornyl acetate (7.4%) and camphene (5.8%) (Table 2).

Twigs from Sharri National Park were dominated by *β*-phellandrene (34.4%), followed by *α*-pinene (17.7%), *β*-pinene (17.4%), germacrene D (6.5%), bornyl acetate (4.3%), and camphene (3.2%). The order of the twigs samples from Bjeshkët e Nemuna National Park was the same as from previous population: *β*-phellandrene (45.1%), followed by *α*-pinene (16.5%), *β*-pinene (11.0%), germacrene D (7.6%), bornyl acetate (2.6%), and camphene (2.6%) (Table 3). Although in lower amounts than aforementioned major constituents, the average percentage of monoterpene, myrcene in both populations was higher than 1%. On the other hand, average percentage of oxygenated monoterpene, *α*-terpineol, in samples from all population originated from National Park “Sharri” was high (8.16% in needles and 3% in twigs) whereas its concentration in the three populations from the National Park “Bjeshkët e Nemuna” was less than 0.2%. Exceptions were the samples from one population (Roshkodol) with percentages reaching 13.5% in needles and 2.1% in twigs.

Experimental results from our study, concerning the composition of essential oils, are in accordance with previously published data. According to Karapandzova et al. [23] the most abundant constituents in samples obtained from* P. peuce* growing in Pelister (Macedonia), in twigs with needles and twigs without needles, were the monoterpenes *α*-pinene (23.8–39.9% and 21.2–23.3%, resp.), camphene, *β*-pinene, myrcene, limonene-phellandrene and bornyl acetate, and the sesquiterpenes, (E)-caryophyllene, germacrene D (7.1–9.5%, 5.0–10.3%), and *δ*-cadinene [2]. Nikolić et al. [18] identified 87 compounds from needle samples collected in locations Ošljak and Pelister, the major constituents of which were *α*-pinene (45.5%), germacrene D (11.1%), *β*-pinene (10.8%), camphene (10.3%), bornyl acetate (5.0%), *β*-phellandrene (3.4%), *β*-caryophyllene (3%), and *β*-myrcene (0.9%). Nikolić et al. [20] also reported that the dominant constituents of essential oils obtained from needles of* P. peuce* from three populations from Serbia and Montenegro were *α*-pinene (36.5%) and germacrene D (11.4%), followed by camphene (8.5%), bornyl acetate (6.8%), *β*-pinene (6.8%), *β*-caryophyllene (5.2%), *β*-phellandrene (4.7%), terpinen-4-ol acetate (1.6%), (E)-hex-2-enal, *α*-muurolene, *β*-gurjunene, and *β*-myrcene. Koukos et al. [29] revealed that the composition of essential oils from needles and twigs of* P. peuce* in Northern Greece has a similar pattern of constituents as those documented in our study. The dominant constituents in twig oil were *α*-pinene (7.4%), *β*-pinene (12.5%), *β*-phellandrene (27.0%), *β*-caryophyllene (4.5%), and citronellol (12.5%), whereas the needle oil was rich in *α*-pinene (23.1%), camphene (5.5%), *β*-pinene (22.0%), *β*-phellandrene (6.8%), bornyl acetate (9.7%), *β*-caryophyllene (3.1%), and citronellol (13.4%). The mean oil yield was 2.9% for twigs and 0.6% for needles. For comparison, in our study the yields of essential oils obtained from twigs were notably higher than those obtained from needles. Average yields of essential oils obtained from samples from the Sharri National Park were in needles 0.85–1.1 and in twigs 2–3.3%, whereas yields from the Bjeshket e Nemuna National Park were in needles 0.8–1.5% and in twigs 1.9–2.7%* v/w*. Higher yields of twigs comparing to needles have been documented in works of other authors [2, 29].

**Figure 1:**
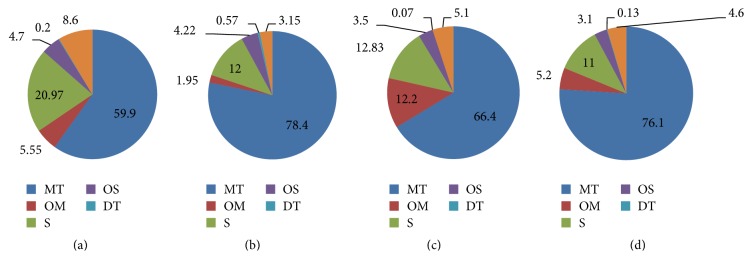
Average concentrations of essential oil constituents obtained from needles (a) and twigs (b) of* Pinus peuce* from Bjeshkët e Nemuna National Park, and needles (c) and twigs (d) from Sharri National Park. MT: monoterpenes; OM: oxygenated diterpenes; S: sesquiterpenes; OS: oxygenated sesquiterpenes; DT: diterpenes. OC: other compounds.

In Figure 1, average concentrations of essential oil constituents obtained from needles (a) and twigs (b) of* Pinus peuce* from Bjeshket e Nemuna National Park and Sharri National Park are presented.

**Figure 2:**
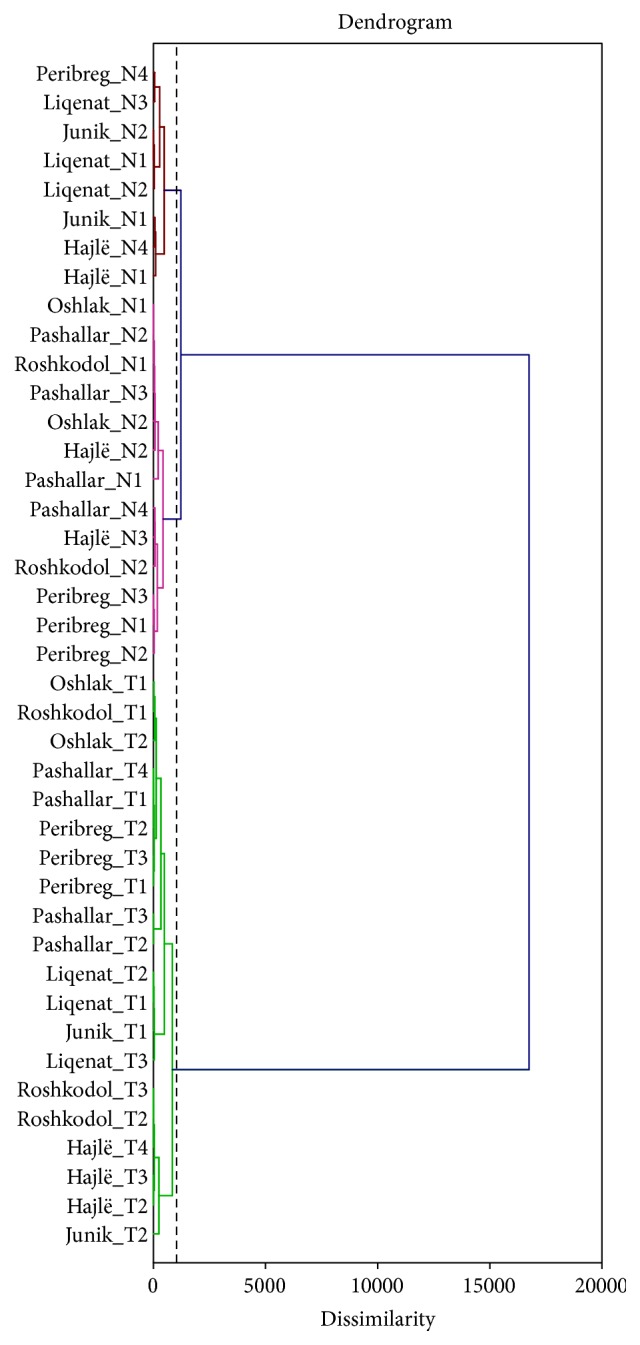
Two-dimensional dendrogram obtained by the cluster analysis of the essential oils of seven populations of* Pinus peuce* based on the unweighed pair group method (square Euclidean distance). N: needles and T: twigs.

**Figure 3:**
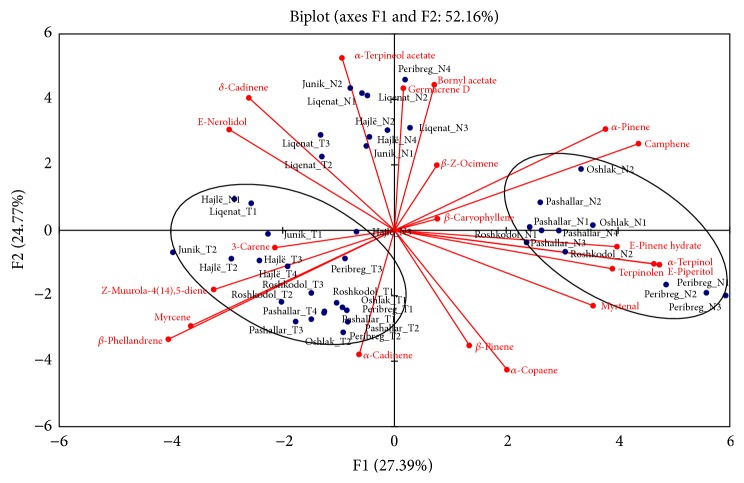
Principal component analysis of the essential oil constituents obtained from needles and twigs of* Pinus peuce.* N: needle and T: twigs.

In order to assess the chemical composition of* P. peuce* essential oils, Hierarchical Cluster Analysis HCA (Figure 2) and Principal component analysis PCA (Figure 3) were performed. The essential oil components with concentrations higher than 2% of the total oil were subjected to statistical analyses. The dendrogram generated from the Euclidean distances performed on the essential oils compounds obtained from needles and twigs of* P. peuce* showed the existence of three main clusters. The first cluster is a group of essential oils obtained from twigs, while the second and third groups cluster samples obtained from needles, suggesting that larger differences in chemical composition were found between the plant organs (needles and twigs).

We also aimed to assess natural variability between the populations. PCA results with few exceptions corresponded with data generated from HCA. The two-dimensional axial system generated from PCA (Figure 3) of essential oils compounds obtained from needles and twigs of* Pinus peuce* shows that there are three main groups. Thus, 3-carene, Z-muurola-4(14),5-diene, myrcene, *β*-phellandrene, and *α*-cadinene were the principal components that contributed to the clustering of the samples obtained from needles. E-nerolidol, *δ*-cadinene, *α*-terpinyl acetate, germacrene D, bornyl acetate, and *β*-Z-ocimene were the primary components that contributed to the clustering of the samples obtained from needles of Junik, Hajlë, Liqenat, and Peribreg and twigs from a sample originated from Hajlë and a sample from Liqenat location. The samples obtained from needles of the locations Oshlak, Pashallar, Roshkodol, and Peribreg were dominated by *α*-pinene, *β*-caryophyllene, camphene, E-pinene hydrate, terpinolene, *α*-terpineol, E-piperitol, and myrtenal (Figure 2).

The PCA results showed that the first two principal axes represented 52% of the total variance and the first axis contributed with 27% of the total variation, while the second axis contributed with 25% of the total variance (Figure 3).

The biggest differences regarding the chemical composition of the essential oil were found among plant organs. This is not surprising because different plant organs have completely different gene expression profiles adapted to the function of the respective organ. Small differences between the populations tested possibly indicate a high genetic relationship among the populations. The significant interaction between populations and plant organs, however, is probably an indication for an environmental influence on gene expression profiles.

HCA and PCA statistical analyses indicate the existence of three groups. The first group of clustered samples was obtained from twigs at all locations with the exception of two samples from the Hajlë site, which were obtained from needles, while the second and third grouped cluster samples were obtained from needles. While the samples obtained from needles were grouped into two clusters, their separation was not based on their origin as we suspected. The samples used in this study were collected in two Kosovar National Parks (Sharri and Bjeshkët e Nemuna National Parks), which are geographically separated from each other at a distance of approximately 100 km. Due to geographic isolation of the mountainous areas and anemophilous pollination of the* P. peuce*, we expected to find two distinct groups based on their chemical composition of essential oils. Statistical analyses of our results did not support this hypothesis, however, as some of our samples from Sharr and Bjeshkët e Nemuna National Park were grouped together. The spatial distribution of the populations suggests that their clustering is not related to their geographic location alone but rather may be linked to local selective forces acting on chemotype diversity. Realistically, the two studied populations represent remnants of a wider and older population and it is not surprising to observe similarities in their respective compositions since this geographic separation occurred in recent times. Low variability related to geographic location is of economic importance since samples originating from different locations in Kosovo can be treated with the same standards.

## 4. Conclusion

In this study, the chemical variability of essential oils obtained from needles and twigs of* Pinus peuce* Griseb. growing in seven locations in Kosovo has been elucidated. Experimental results revealed that the dominant constituents of oils were monoterpenes, whereas sesquiterpenes and diterpenes were present in lower amounts. *α*-Pinene, *β*-phellandrene, *β*-pinene, germacrene D, and bornyl acetate were dominant constituents of the essential oils. Statistical analysis of PCA and HCA documented that variability exists in the composition of essential oils and that this is primarily related to the plant organ source of the essential oil, rather than interspecies variation between different populations. Variability in chemical composition of essential oils among populations of* P. peuce* seems to reflect the environmental impact on these compositions, which is influenced by differences in microclimatic conditions. To confirm the natural variability and chemopolymorphism of this species in Kosovo, further investigation is warranted and should be corroborated with more comprehensive molecular analysis.

## Figures and Tables

**Table 1:** Basic characterization of the sites from where the plant materials of *Pinus peuce* populations were collected.

Locations	North	South	Elevation m.a.s.l.	Herbarium accession number
Peribreg^a^	42°10′42′′	21°01′51′′	1717	LEB 2013/10
Oshlak^a^	42°10′57′′	20°56′52′′	1477	LEB 2013/9
Pashallar^a^	42°15′01′′	20°54′55′′	1644	LEB 2013/12
Junik^b^	42°30′25′′	20°12′47′′	1374	LEB 2013/14
Liqenat^b^	42°40′11′′	20°05′41′′	1870	LEB 2013/13
Hajlë^b^	42°44′44′′	20°07′37′′	1764	LEB 2013/8
Roshkodol^b^	42°37′44′′	20°06′21′′	1728	LEB 2013/11

^a^Sharri national park; ^b^ Bjeshkët e Nemuna National Park.

**Table 2:** Composition (%) of the needles oils of *Pinus peuce* from different locations.

			Sharri National Park				Bjeshkët e Nemuna National Park				
RT	KI	Compounds	Peribreg	Oshlak	Pashallar	Average	Junik	Liqenat	Hajlë	Roshkodol	Average
7.80	926	Tricyclene	0.5	0.5	0.5	0.5	0.3	0.5	0.2	0.5	0.37
*8.16*	*940*	*α-Pinene*	*29.1*	*31.0*	*34.7*	*31.6*	*26.7*	*33.0*	*21.6*	*34.9*	*29.0*
*8.58*	*954*	*Camphene*	*8.3*	*7.4*	*7.4*	*7.7*	*5.1*	*6.9*	*2.7*	*8.7*	*5.8*
9.26	975	Sabinene	0.03	0.05	0.03	0.04	0.04	0.1	0.1	0.01	0.06
*9.43*	*979*	*β-Pinene*	*16.1*	*11.8*	*13.6*	*13.8*	*10.3*	*12.2*	*10.0*	*11.3*	*10.9*
*9.68*	*990*	*Myrcene*	*0.6*	*1.8*	*0.8*	*1.1*	*0.7*	*0.9*	*1.3*	*0.7*	*0.9*
10.22	1005	*α*-Phellandrene	0.2	0.1	0.2	0.2	0.2	0.15	0.2	0.2	0.2
*10.42*	*1011*	*3-Carene*	*0.02*	*0.15*	*0.2*	*0.1*	*0.2*	*0.4*	*0.9*	*0.00*	*0.4*
10.87	1017	*α*-Terpinene	0.07	0.07	0.07	0.1	0.07	0.05	0.07	0.07	0.06
*11.10*	*1029*	*β-Phellandrene*	*5.7*	*19.5*	*4.1*	*9.8*	*8.2*	*4.6*	*27.7*	*5.1*	*11.4*
*13.08*	*1037*	*β-Z-Ocimene*	*0.1*	*0.2*	*1.0*	*0.4*	*0.4*	*0.5*	*0.4*	*0.01*	*0.3*
14.30	1059	*γ*-Terpinene	0.3	0.2	0.5	0.3	0.4	0.15	0.2	0.5	0.3
*14.94*	*1088*	*Terpinolene*	*1.5*	*0.1*	*0.3*	*0.6*	*0.2*	*0.04*	*0.2*	*0.1*	*0.13*
*15.00*	*1122*	*E-Pinene hydrate*	*1.3*	*0.3*	*0.3*	*0.6*	*0.3*	*0.2*	*0.2*	*0.2*	*0.2*
15.97	1126	*α*-Campholenal	0.4	0.3	0.1	0.3	0.6	0.7	0.6	0.1	0.5
16.36	1138	iso-3-Thujanol	0.6	0.4	0.7	0.6	0.1	0.1	0.1	0.8	0.3
16.82	1141	Z-Verbenol	0.2	0.2	0.4	0.3	0.2	0.2	0.2	0.3	0.2
17.09	1165	Borneol	0.7	0.2	0.1	0.3	0.3	0.2	0.2	0.1	0.2
17.42	1177	Terpinene-4-ol	0.1	0.01	0.04	0.0	0.1	0.1	0.1	0.1	0.1
*17.59*	*1188*	*α-Terpineol*	*8.8*	*5.8*	*9.8*	*8.1*	*0.1*	*0.1*	*0.1*	*13.5*	*3.4*
*17.82*	*1195*	*Myrtenal*	*1.0*	*0.2*	*0.2*	*0.5*	*0.2*	*0.05*	*0.1*	*0.2*	*0.1*
*17.89*	*1208*	*E-Piperitol*	*1.1*	*1.2*	*1.5*	*1.3*	*0.0*	*0.03*	*0.01*	*1.6*	*0.4*
18.25	1216	E-Carveol	0.0	0.07	0.02	0.0	0.04	0.0	0.05	0.02	0.0
18.78	1257	Linalool acetate	0.1	0.1	0.8	0.1	0.05	0.01	0.08	0.07	0.1
*20.32*	*1285*	*Bornyl acetate*	*5.9*	*4.2*	*3.0*	*4.4*	*11.0*	*9.6*	*5.9*	*3.3*	*7.4*
*22.49*	*1349*	*α-Terpineol acetate*	*0.7*	*0.2*	*0.2*	*0.4*	*2.0*	*1.6*	*1.0*	*0.02*	*1.1*
*23.58*	*1376*	*α-Copaene*	*0.4*	*0.3*	*0.4*	*0.4*	*0.1*	*0.1*	*0.1*	*0.5*	*0.2*
23.94	1384	*β*-Bourbonene	0.2	0.1	0.2	0.2	0.3	0.2	0.1	0.2	0.2
24.07	1391	*β*-Elemene	0.04	0.1	0.0	0.0	0.2	0.2	0.2	0.0	0.1
*25.45*	*1418*	*β-Caryophyllene*	*0.0*	*0.03*	*2.1*	*0.7*	*0.2*	*0.1*	*0.1*	*0.0*	*0.1*
26.07	1432	*β*-Copaene	0.1	0.7	0.2	0.3	0.2	0.3	0.2	0.1	0.2
26.31	1434	*α*-E-Bergamotene	0.2	0.2	0.0	0.1	0.8	0.7	0.7	0.0	0.5
26.60	1454	*α*-Humulene	0.05	0.1	0.1	0.1	0.2	0.2	0.2	0.0	0.1
*27.00*	*1466*	*Z-Muurola-4(14),5-diene*	*0.2*	*0.2*	*0.3*	*0.2*	*0.6*	*0.4*	*0.6*	*0.0*	*0.4*
*27.25*	*1481*	*Germacrene D*	*9.2*	*8.1*	*10.2*	*9.2*	*22.1*	*19.3*	*14.5*	*10.5*	*16.6*
27.42	1500	Bicyclogermacrene	0.5	0.4	0.8	0.6	0.1	0.1	0.1	0.8	0.3
27.61	1513	*γ*-Cadinene	0.1	0.1	0.1	0.1	0.2	0.2	0.3	0.1	0.2
*27.73*	*1523*	*δ-Cadinene*	*0.5*	*0.3*	*0.3*	*0.4*	*1.5*	*1.3*	*1.3*	*0.3*	*1.1*
*28.24*	*1538*	*α-Cadinene*	*0.7*	*0.6*	*0.8*	*0.7*	*0.5*	*0.3*	*0.9*	*1.2*	*0.7*
*28.49*	*1563*	*E- Nerolidol*	*0.3*	*0.6*	*0.03*	*0.3*	*1.3*	*1.1*	*2.1*	*0.0*	*1.1*
29.57	1574	Germacrene-4-ol	0.8	0.1	0.8	0.6	0.5	0.7	0.3	0.1	0.4
30.26	1576	Spathulenol	0.2	0.2	0.4	0.3	0.05	0.1	0.3	0.1	0.1
30.36	1583	Caryophyllene oxide	0.4	0.2	0.1	0.2	0.5	0.3	0.5	0.4	0.4
31.39	1636	Humulene epoxide II	0.3	0.3	0.3	0.9	0.4	0.2	0.4	0.3	0.3
32.08	1636	Z-Cadin-4-en-7ol	0.3	0.2	0.5	0.3	0.0	0.0	0.1	0.5	0.1
32.28	1646	Cubenol	0.1	0.1	0.0	0.1	0.5	0.5	0.6	0.0	0.4
32.40	1646	*α*-Muurolol	0.3	0.1	0.2	0.2	0.2	0.1	0.2	0.2	0.2
32.69	1653	*α*-Cadinol	0.5	0.4	0.31	0.4	0.9	0.8	1.0	0.3	0.7
33.68	1688	Eudesma-4(15),7-diene-1-*β*-ol	0.3	0.2	0.3	0.3	0.3	0.2	0.2	0.7	0.3
38.96	1733	Oplopanone	0.6	0.2	0.6	0.5	0.2	0.1	0.3	1.0	0.4
45.69	1929	Cembrene	0.05	0.03	0.1	0.1	0.1	0.0	0.4	0.1	0.1

		Yield % *v*/*w*	0.8–1.5	0.7–1.0	0.7–1.0		1.2–1.4	0.8–1.2	0.7–0.9	1.3–1.7	

		Monoterpenes	62.6	73.1	63.4		52.7	59.4	65.5	62.2	
		Oxygenated monoterpenes	14.3	9.0	13.2		2.0	1.6	1.7	16.9	
		Sesquiterpenes	12.2	10.7	15.6		27.1	23.5	19.5	13.8	
		Oxygenated sesquiterpenes	4.1	2.7	3.7		4.9	4.4	5.9	3.6	
		Diterpenes	0.1	0.0	0.1		0.1	0.0	0.4	0.1	
		Others	6.7	4.6	3.9		13.0	11.2	7.0	3.3	

**Table 3:** Composition (%) of the twigs oils of *Pinus peuce* from different locations.

			Sharri National Park				Bjeshkët e Nemuna National Park				
RT	KI	Compounds	Peribreg	Oshlak	Pashallar	Average	Junik	Liqenat	Hajlë	Roshkodol	Average
7.80	926	Tricyclene	0.4	0.1	0.1	0.2	0.1	0.4	0.1	0.1	0.17
*8.16*	*940*	*α-Pinene*	*24.0*	*11.0*	*18.0*	*17.7*	*14.9*	*20.2*	*16.5*	*14.3*	*16.5*
*8.58*	*954*	*Camphene*	*5.9*	*1.6*	*2.1*	*3.2*	*1.9*	*5.1*	*1.3*	*2.1*	*2.6*
9.26	975	Sabinene	0.03	0.00	0.03	0.02	0.10	0.05	0.1	0.03	0.07
*9.43*	*979*	*β-Pinene*	*12.0*	*20.7*	*19.6*	*17.4*	*6.9*	*9.7*	*11.1*	*16.5*	*11.0*
*9.68*	*990*	*Myrcene*	*1.8*	*1.4*	*2.4*	*1.9*	*2.8*	*1.4*	*1.5*	*1.3*	*1.7*
10.22	1005	*α*-Phellandrene	0.1	0.1	0.1	0.1	0.05	0.25	0.1	0.04	0.1
*10.42*	*1011*	*3-Carene*	*0.1*	*0.00*	*1.6*	*0.6*	*1.3*	*0.1*	*0.9*	*0.1*	*0.6*
10.87	1017	*α*-Terpinene	0.07	0.06	0.08	0.1	0.03	0.08	0.08	0.04	0.06
*11.10*	*1029*	*β-Phellandrene*	*29.0*	*40.4*	*34.*	*34.5*	*49.8*	*34.0*	*48.5*	*48.2*	*45.1*
*13.08*	*1037*	*β-Z-Ocimene*	*0.15*	*0.0*	*0.0*	*0.0*	*0.3*	*0.2*	*0.2*	*0.02*	*0.18*
14.30	1059	*γ*-Terpinene	0.2	0.2	0.3	0.2	0.0	0.1	0.1	0.2	0.1
*14.94*	*1088*	*Terpinolene*	*0.2*	*0.2*	*0.2*	*0.2*	*0.0*	*0.2*	*0.1*	*0.04*	*0.1*
*15.00*	*1122*	*E-Pinene hydrate*	*0.2*	*0.05*	*0.03*	*0.1*	*0.1*	*0.3*	*0.2*	*0.05*	*0.16*
15.97	1126	*α*-Campholenal	0.6	0.5	0.4	0.5	0.3	0.3	0.3	0.3	0.3
16.36	1138	iso-3-Thujanol	0.3	0.2	0.3	0.3	0.1	0.2	0.1	0.2	0.15
16.82	1141	Z-Verbenol	0.2	0.2	0.2	0.2	0.2	0.2	0.2	0.2	0.2
17.09	1165	Borneol	0.2	0.1	0.07	0.1	0.1	0.3	0.2	0.04	0.16
17.42	1177	Terpinene-4-ol	0.1	0.2	0.1	0.1	0.0	0.05	0.0	0.2	0.1
*17.59*	*1188*	*α-Terpineol*	*2.9*	*2.7*	*3.4*	*3.1*	*0.04*	*0.1*	*0.1*	*2.1*	*0.6*
*17.82*	*1195*	*Myrtenal*	*0.4*	*0.3*	*0.3*	*0.3*	*0.1*	*0.2*	*0.3*	*0.1*	*0.2*
*17.89*	*1208*	*E-Piperitol*	*0.3*	*0.3*	*0.4*	*0.3*	*0.0*	*0.1*	*0.0*	*0.1*	*0.0*
18.25	1216	E-Carveol	0.03	0.03	0.2	0.1	0.03	0.1	0.1	0.3	0.1
18.78	1257	Linalool acetate	0.1	0.1	0.1	0.1	0.05	0.08	0.20	0.08	0.1
*20.32*	*1285*	*Bornyl acetate*	*7.4*	*2.5*	*3.0*	*4.3*	*1.8*	*5.6*	*1.3*	*1.8*	*2.6*
*22.49*	*1349*	*α-Terpineol acetate*	*0.4*	*0.1*	*0.1*	*0.2*	*0.2*	*1.2*	*0.2*	*0.05*	*0.4*
*23.58*	*1376*	*α-Copaene*	*0.5*	*0.5*	*0.5*	*0.5*	*0.1*	*0.1*	*0.2*	*0.4*	*0.2*
23.94	1384	*β*-Bourbonene	0.2	0.6	0.4	0.4	0.0	0.1	0.01	0.4	0.1
24.07	1391	*β*-Elemene	0.04	0.0	0.0	0.0	0.1	0.2	0.2	0.0	0.1
*25.45*	*1418*	*β-Caryophyllene*	*0.03*	*0.0*	*0.0*	*0.0*	*0.1*	*0.3*	*0.1*	*0.0*	*0.1*
26.07	1432	*β*-Copaene	0.1	0.1	0.1	0.1	0.1	0.2	0.1	0.05	0.1
26.31	1434	*α*-E-Bergamotene	0.1	05	0.6	0.4	0.6	0.4	0.5	0.0	0.4
26.60	1454	*α*-Humulene	0.1	0.1	0.5	0.2	0.2	0.9	0.1	0.0	0.3
*27.00*	*1466*	*Z-Muurola-4(14),5-diene*	*1.0*	*1.0*	*0.9*	*1.0*	*0.5*	*0.4*	*0.8*	*0.6*	*0.6*
*27.25*	*1481*	*Germacrene D*	*6.3*	*8.3*	*4.9*	*6,5*	*10.6*	*7.8*	*6.7*	*5.4*	*7.6*
27.42	1500	Bicyclogermacrene	0.2	0.3	0.4	0.3	0.1	0.3	0.1	0.5	0.2
27.61	1513	*γ*-Cadinene	0.1	0.2	0.1	0.1	0.2	0.4	0.3	0.1	0.2
*27.73*	*1523*	*δ-Cadinene*	*0.5*	*0.5*	*0.5*	*0.5*	*1.0*	*1.5*	*0.7*	*0.6*	*1.0*
*28.24*	*1538*	*α-Cadinene*	*0.7*	*1.1*	*1.0*	*0.9*	*0.8*	*0.5*	*1.0*	*1.3*	*1.0*
*28.49*	*1563*	*E- Nerolidol*	*0.2*	*0.0*	*0.0*	*0.1*	*1.6*	*3.1*	*2.1*	*0.0*	*1.7*
29.57	1574	Germacrene-4-ol	0.4	0.3	0.1	0.3	0.1	0.4	0.2	0.1	0.2
30.26	1576	Spathulenol	0.0	0.1	0.2	0.1	0.3	0.4	0.1	0.0	0.2
30.36	1583	Caryophyllene oxide	0.3	0.2	0.2	0.2	0.1	0.1	0.3	0.2	0.2
31.39	1636	Humulene epoxide II	0.2	0.2	0.2	0.6	0.4	0.4	0.5	0.3	0.4
32.08	1636	Z-Cadin-4-en-7ol	0.5	0.6	0.6	0.6	0.2	0.1	0.1	0.4	0.2
32.28	1646	Cubenol	0.1	0.0	0.0	0.0	0.3	0.7	0.3	0.0	0.3
32.40	1646	*α*-Muurolol	0.4	0.4	0.3	0.4	0.1	0.2	0.1	0.3	0.2
32.69	1653	*α*-Cadinol	0.2	0.5	0.2	0.3	0.5	0.5	0.6	0.1	0.4
33.68	1688	Eudesma-4(15),7-diene-1-*β*-ol	0.2	0.2	0.3	0.2	0.1	0.1	0.2	0.2	0.1
38.96	1733	Oplopanone	0.3	1.1	0.6	0.7	0.0	0.2	0.0	0.5	0.2
45.69	1929	Cembrene	0.05	0.2	0.1	0.1	0.9	0.2	0.5	0.1	0.4

		Yield % *v*/*w*	2.3–3.2	3.0–3.3	1.7–2.3		2.4–2.6	1.6–2.2	1.8–2.0	2.4–3.0	

		Monoterpenes	74.0	75.8	78.5		78.3	71.8	80.5	82.9	
		Oxygenated monoterpenes	5.3	4.7	5.6		0.9	1.7	1.5	3.7	
		Sesquiterpenes	9.9	13.2	9.9		14.5	13.1	11.1	9.3	
		Oxygenated sesquiterpenes	2.9	3.5	2.8		3.8	6.3	4.7	2.1	
		Diterpenes	0.1	0.2	0.1		0.9	0.2	0.5	0.1	
		Others	7.9	2.6	3.2		2.1	6.9	1.7	1.9	

## Abbreviations

ESI-MS: Electrospray Ionization Mass Spectrometry
GC: Gas Chromatography
GC-FID: Gas Chromatography-Flame Ionization Detection
GC-MS: Gas Chromatography-Mass Spectrometry
HCA: Hierarchical Cluster Analysis
HPLC: High performance liquid chromatography
PCA: Principal Component Analyses.
